# Serum apolipoprotein C3 levels are negatively associated with hepatitis B virus DNA in HBeAg-negative chronic hepatitis B patients

**DOI:** 10.1186/s12944-019-1084-6

**Published:** 2019-06-11

**Authors:** Yu Cui, Xiang-Dan Cui, Meng Xu, Min Fang, Mei-Juan Cai

**Affiliations:** 1 Department of Obstetrics, Yantai Yuhuangding Hospital, Qingdao University, Yantai, China; 2 Department of Gastroenterology, Qilu Hospital of Shandong University, Jinan, China; 3 Department of Clinical Laboratory, Qilu Hospital of Shandong University, No. 107 Wenhua Xi Road, Jinan, 250012 Shandong China; 4 Department of Gynaecology, Qingdao Women’s and Children’s Hospital, Qingdao University, Qingdao, China

**Keywords:** Hepatitis B virus, HBeAg, ApoC3, ApoA5

## Abstract

**Background:**

Hepatitis B virus (HBV) infection remains a global health issue associated with substantial morbidity and mortality. Serum apolipoprotein C3 (ApoC3) and apolipoprotein A5 (ApoA5) levels were decreased in chronic hepatitis B (CHB) patients, however the relationship between ApoC3 or ApoA5 and HBV DNA load remains elusive.

**Methods:**

A total of 384 CHB patients including 194 HBsAg(+) HBeAg(−) and 190 HBsAg(+) HBeAg(+) and 154 healthy individuals were recruited in our study. Serum levels of alanine aminotransferase (ALT), aspartate transaminase (AST), total cholesterol (Chol), triglycerides (TG), apolipoprotein A1 (ApoA1), apolipoprotein B (ApoB), high-density lipoproteins cholesterol (HDL-C), low-density lipoproteins cholesterol (LDL-C) and lipoprotein a (Lpa) were examined in an automatic biochemical analyzer. Apolipoprotein A5 (ApoA5) and apolipoprotein C3 (ApoC3) were detected via ELISA.

**Results:**

Serum ApoA1, ApoB, ApoC3 and ApoA5 levels were reduced in CHB patients. In HBeAg(−) CHB patients, plasma ApoC3 levels were negatively associated with HBV DNA load (r = 0.219, *P* < 0.001). But no correlation between ApoA5 and HBV DNA load was observed in CHB patients.

**Conclusions:**

These data showed that HBV infection inhibits lipid metabolism and ApoC3 is negatively associated with HBV DNA load in HBeAg (−) CHB patients. These findings provided new evidence about the link between ApoC3-related lipid metabolism and immune response.

## Introduction

Liver is the key organ for lipoproteins synthesis, degradation and lipid metabolism [1]. Infection by hepatitis B virus (HBV) remains a global health issue associated with substantial morbidity and mortality [2], which causes various chronic liver diseases, liver cirrhosis and hepatocellular carcinoma [3]. Although some reports were focused on the pathogenesis of chronic hepatitis B (CHB) [4, 5], the relationship between HBV and lipid metabolism remains elusive.

During the process of normal transport, lipids are combined with apolipoprotein (Apo) [6]. ApoA5 and ApoC3 are important members of Apo family. ApoA5 is found to be located in ApoA1/C3/A4 gene cluster on human chromosome 11q23 [7] and is present in high-density lipoprotein cholesterol (HDL-C), very low-density lipoprotein and chylomicrons [8]. ApoC3 is a 79 amino-acid glycoprotein synthesized mainly in the liver [9]. Some reports indicated that ApoC3 plays a central role in regulating the plasma metabolism of TG-rich lipoproteins (TRL), and decreases TRL hydrolysis [5, 10]. ApoC3 and ApoA5 are associated with multiple lipid metabolism-related diseases, such as type 1 diabetes and coronary artery disease [11–13]. Although previous study showed that HBV inhibits ApoA5 and ApoC3 promoter activity and mRNA and protein expression through its core gene [14] or X gene [15], respectively, little was revealed about the association between ApoC3 or ApoA5 and HBV replication rate in CHB patients.

In our study, we mainly detected the serum levels of ApoC3 and ApoA5 in CHB patients and healthy individuals. We found that the serum ApoC3 levels were negatively associated with HBV DNA load in HBeAg-negative CHB patients, and no significant relationship was observed between ApoA5 and HBV DNA load in CHB patients. These findings enriched the function of ApoC3 and provided new insight for the management of anti-viral treatment in HBV clinical practice.

## Materials and methods

### Study subjects

A total of 384 CHB patients and 154 healthy individuals were recruited from Qihu Hospital of Shandong University (Qingdao) between December 2016 and September 2018. There were 288 males and 250 females among 538 individuals. The CHB patients were comprised of 194 HBsAg(+) HBeAg(−) and 190 HBsAg(+) HBeAg(+). CHB was diagnosed based on serum HBsAg (+) for at least 6 months. The patients who had a history of critical organs disease such as heart, brain and other chronic liver diseases that can cause metabolic lipid abnormalities were excluded. The 154 healthy individuals were comprised of sex-matched HBsAg(−) volunteers from the Medical Examination Center of Qilu Hospital of Shandong University (Qingdao). The study was approved by the Ethics Committee of Qilu Hospital of Shandong University (Qingdao) (KYLL-2016(KS)-173, http://www.qiluhospital.com/) and all subjects signed the informed consent.

### Biochemical testing

Blood samples were collected from each individual after overnight fasting. After centrifugation at 3500 rpm for 8 min, sera were collected and stored at 80 °C for follow-up experiments. Serum levels of alanine aminotransferase (ALT, IFCC method), aspartate transaminase (AST, colorimetry), total cholesterol (Chol, enzymatic colorimetry), triglycerides (TG, colorimetry), total bilirubin (TBI, Diazo method) and direct bilirubin (DBI, Diazo method) were assessed using Roche Cobas c 701 (Roche, Switzerland). The levels of apolipoprotein A1 (ApoA1) and apolipoprotein B (ApoB) were examined using immunoturbidimetric method, and the serum levels of high-density lipoproteins cholesterol (HDL-C), low-density lipoproteins cholesterol (LDL-C) were detected using surfactant clearance method, and lipoprotein a (Lpa) was examined using latex immunoturbidimetry method in Roche Cobas c 701 (Roche, Switzerland).

### Virological assessment

Serum levels of HBsAg and HBeAg were measured using Roche Cobas e 601 (Roche, Switzerland). HBV DNA was examined with Hepatitis B Viral Quantitative Fluorescence Diagnosis Kit (Sansure Biotech Inc., Hunan, China) by using Real-time Fluorescene Quantitative polymerase chain reaction system LightCycler 480 II (Roche, Switzerland).

### Enzyme-linked immunosorbent assay (ELISA) for ApoC3 and ApoA5

Serum levels of ApoC3 and ApoA5 were assessed with the sandwich ELISA Kits (Jingmei Biological Technology Co.Ltd., Jiangsu, China) according to the protocol. The optical density (OD) value was measured at the wavelength of 450 nm by using Thermo Fisher Multiskan FC photometer (Thermo Fisher Scientific, Waltham, MA).

### Statistical analysis

Data were expressed as means ± standard deviations. Statistical analysis was performed by IBM SPSS Statistics 25.0 (IBM Corporation, USA) and GraphPad Prism 5.01 (GraphPad Software, Inc., California, USA). Differences were compared by a one-way analysis of variance (ANOVA), followed by LSD post hoc test. Relationship between two variables was determined by Pearson’s correlation coefficient. Two tailed *P* < 0.05 was considered statistically significant.

## Results

### Baseline characteristics

The clinical and liver biochemical profiles of participants in the study cohort were listed in Table 1. There were 538 individuals in the study, containing 154 healthy volunteers, 194 HBeAg(−) HBsAg(+) and 190 HBeAg (+) HBsAg(+) patients. No significant differences in gender, age and BMI were observed among Healthy, HBeAg (−) and HBeAg (+) groups (*P* > 0.05). The serum ALT levels in HBeAg(−) and HBeAg(+) groups were significantly higher than those in Healthy group (*P* < 0.01), and the level of ALT in HBeAg(+) displayed the highest. Similarly, compared with Healthy group, the levels of AST in HBeAg(−) and HBeAg(+) were enhanced (P < 0.01), however there was no significant difference between HBeAg(−) and HBeAg(+) groups (*P* > 0.05). No significant difference on TBI levels was observed between healthy and HBeAg(+) groups (P > 0.05), but the serum TBI level in HBeAg(−) group was higher than that in Healthy group (*P* < 0.01). Compared with Healthy group, the levels of DBI in HBeAg(−) and HBeAg(+) groups were significantly higher than those in Healthy group (*P* < 0.01, *P* < 0.05). Log HBV DNA in HBeAg(+) group was higher than that in HBeAg(−) group (P < 0.01).

**Table 1:** Clinical and liver biochemical profiles of participants in the study

Groups	Healthy (*n* = 154)	HBeAg-negative (*n* = 194)	HBeAg-positive (*n* = 190)
Gender (M/F)	58/96	104/90	92/98
Age (years) BMI (kg/m^2^)	45.31 ± 14.84 21.91 ± 1.01	43.97 ± 14.05 22.11 ± 1.82	39.93 ± 12.98 22.04 ± 1.58
ALT (U/L)	15.52 ± 0.76	39.72 ± 5.54**	62.16 ± 8.34**^##^
AST (U/L)	17.09 ± 0.45	40.87 ± 4.16**	48.39 ± 5.00**
TBI (μM)	11.20 ± 0.53	11.27 ± 0.75**	11.78 ± 0.85
DBI (μM)	4.37 ± 0.18	5.73 ± 1.02**	4.87 ± 0.62*
Log HBV DNA		3.82 ± 1.70	6.25 ± 1.90^##^

*P < 0.05, **P < 0.01, compared with Healthy group; ^##^*P* < 0.01, compared with HBeAg-negative group. BMI, Body mass index

### The plasma levels of TG, HDL-C and LDL-C were decreased in CHB patients

**Fig. 1:**
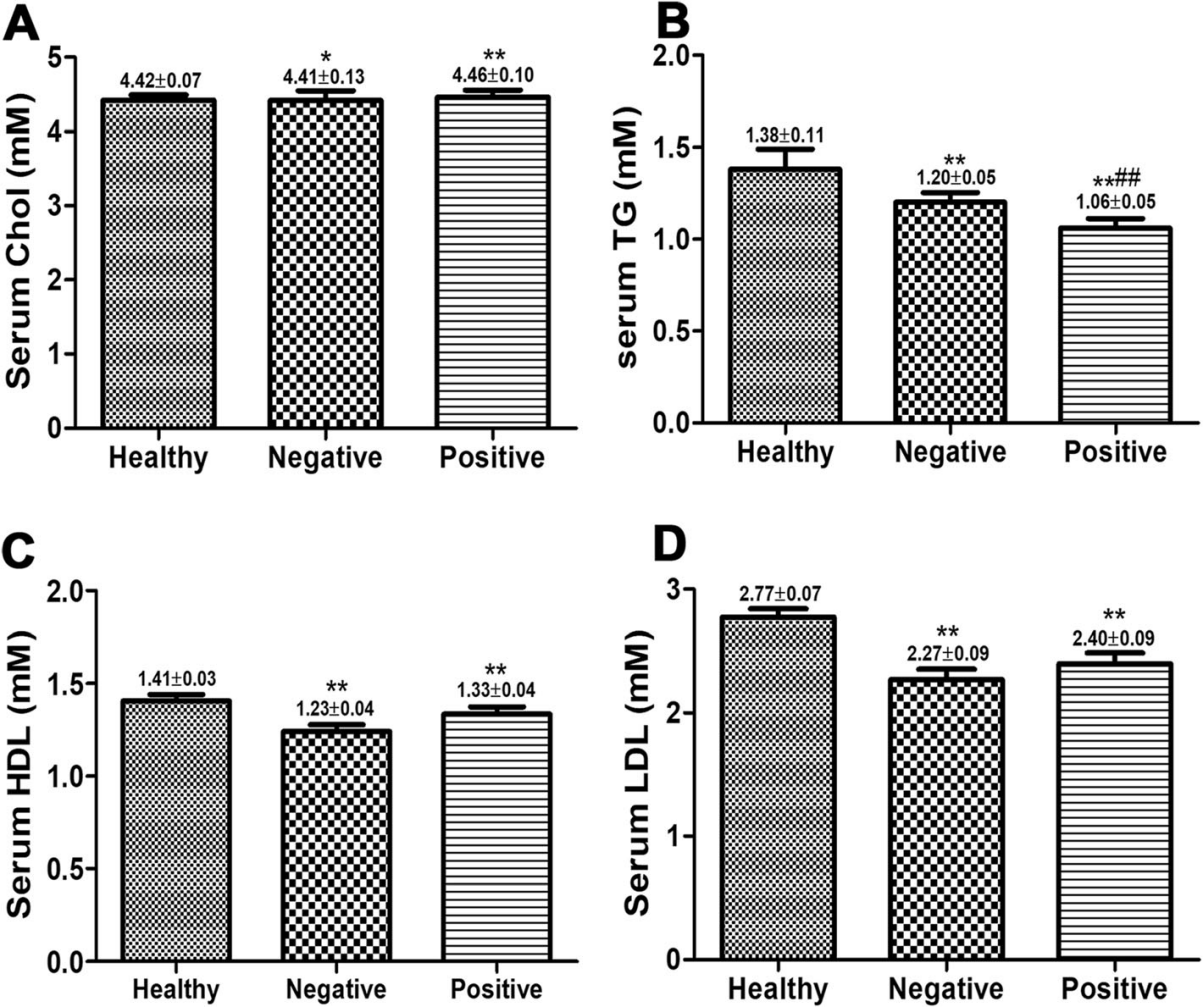
Serum TG, HDL-C and LDL-C levels were blocked in CHB patients. CHB patients exhibited elevated serum Chol levels (**a**) and decreased TG levels (**b**), HDL-C levels (**c**) and LDL-C levels (**d**). Compared with Healthy group, *P < 0.05, **P < 0.01; Compared with Negative group, #*P* < 0.05, ##*P* < 0.01

We further investigated the levels of Chol, TG, HDL-C and LDL-C in Healthy, HBeAg(−) and HBeAg(+) groups. As shown in Fig. 1a, compared with Healthy group, the levels of Chol in HBeAg(−) and HBeAg(+) groups were significantly enhanced (*P* < 0.05, *P* < 0.01), and no significant difference was observed between HBeAg(−) and HBeAg(+) groups (*P* > 0.05). In Fig. 1b, the serum TG in Healthy group was the highest, and the serum TG in HBeAg(+) group was significantly lower than that in HBeAg(−) (*P* < 0.01). Compared with healthy group, the serum HDL-C and LDL-C levels in both HBeAg(−) and HBeAg(+) groups were significantly reduced (*P* < 0.01), and there was no significant difference between HBeAg(−) and HBeAg(+) groups (*P* > 0.05).

### The serum ApoA1, ApoB, ApoA5 and ApoC3 levels were reduced in CHB patients

**Fig. 2:**
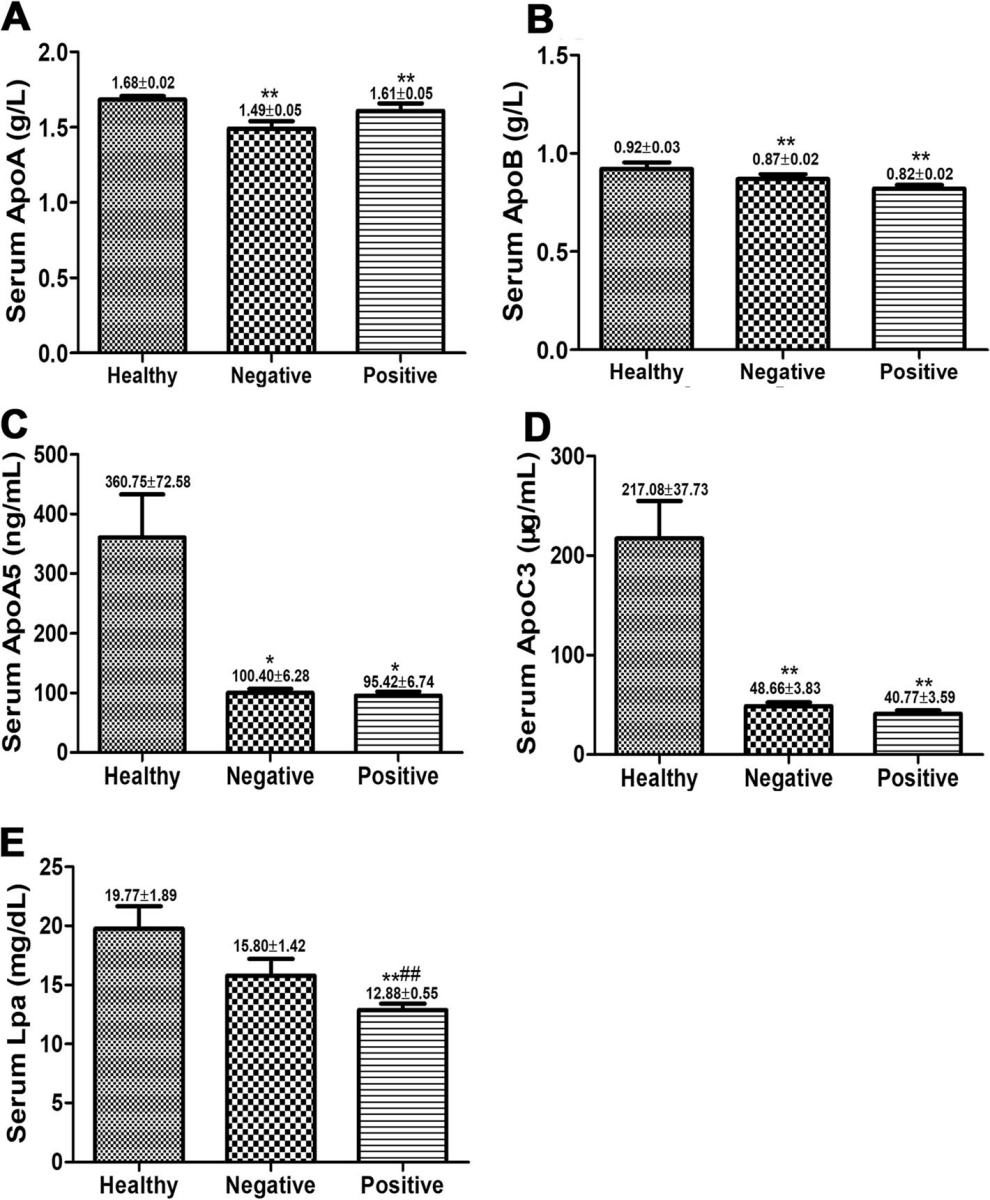
Serum ApoA1, ApoB, ApoA5, ApoC3 and Lpa levels were decreased in CHB patients. Compared with Healthy group, ApoA1 (**a**), ApoB (**b**), ApoA5 (**c**), ApoC3 (**d**) and Lpa (**e**) levels in CHB patients were significantly blocked. Compared with Healthy group, *P < 0.05, **P < 0.01; Compared with Negative group, #*P* < 0.05, ##*P* < 0.01

As shown in Fig. 2a-d, the serum levels of ApoA1, ApoB, ApoA5 and ApoC3 in HBeAg(−) and HBeAg(+) groups were significantly blocked than those in Healthy group (*P* < 0.01, P < 0.01, *P* < 0.05, P < 0.01), and no significant differences between HBeAg(−) and HBeAg(+) groups were detected (*P* > 0.05). As displayed in Fig. 2e, the serum Lpa level in HBeAg(+) was significantly lower than that in Healthy group (*P* < 0.01), and there was no significant difference between Healthy and HBeAg(−) groups (*P* > 0.05).

### Serum ApoC3 levels were negatively associated with HBV DNA load in HBeAg (−) patients

**Fig. 3:**
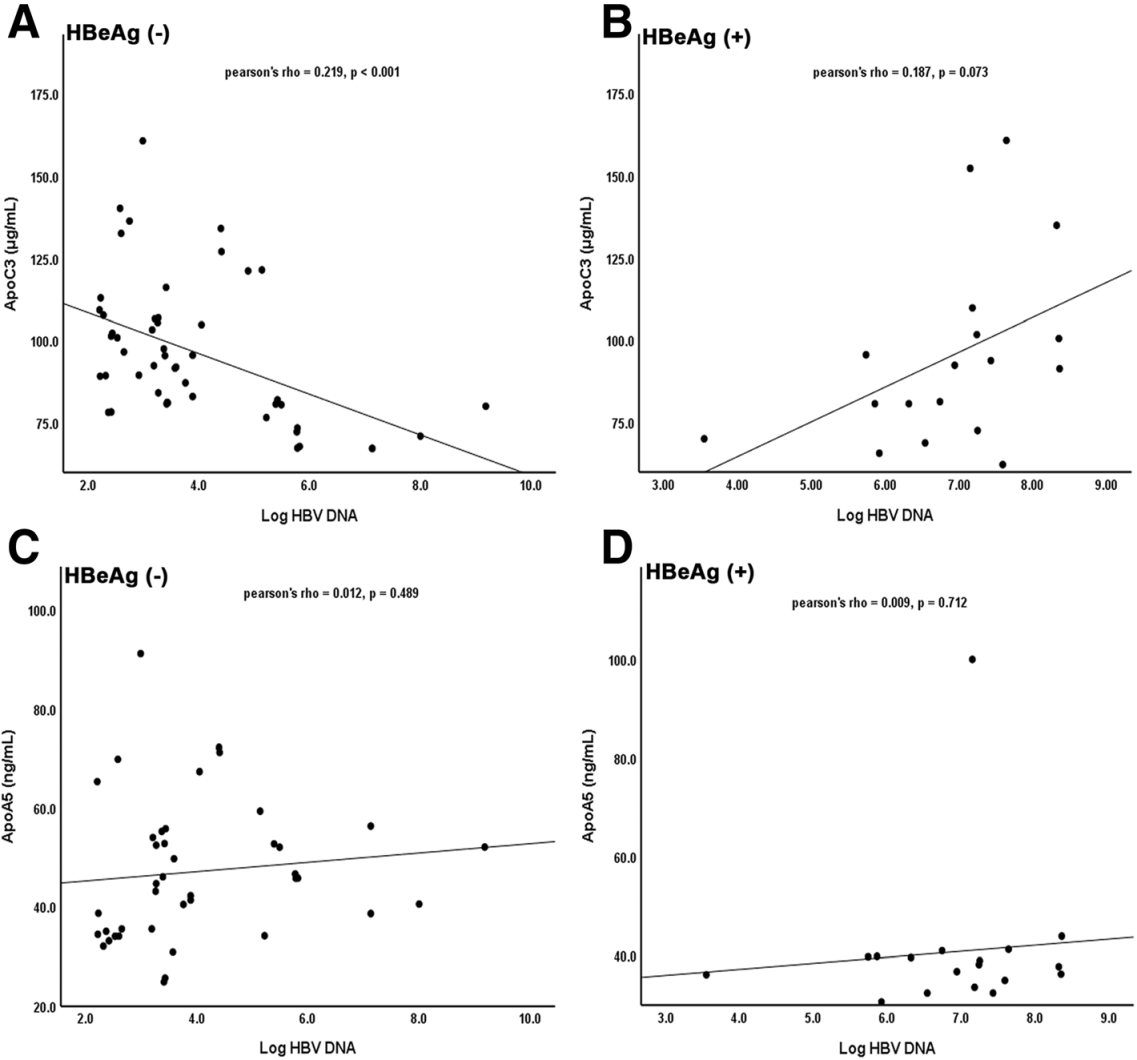
Serum ApoC3 levels were negatively associated with HBV DNA load in HBeAg (−) CHB patients. A and B displayed the relationship between ApoC3 and HBV DNA load in HBeAg (−) and HBeAg (+) CHB patients. C and D exhibited the relationship between ApoA5 and HBV DNA load in HBeAg (−) and HBeAg (+) CHB patients. Relationship between two variables was determined by Pearson’s correlation coefficient

We further investigated the relationship between ApoC3 or ApoA5 and HBV DNA load in CHB patients by using double variables correlation in SPSS software in Fig. 3. As shown in Fig. 3a and b, negative correlation between ApoC3 and HBV DNA load was observed in HBeAg (−) patients (*P* < 0.001), but not in HBeAg (+) patients (*P* = 0.073). In Fig. 3c and d, there was no significant correlation between ApoA5 level and HBV DNA load in HBeAg (−) and HBeAg (+) patients (*P* = 0.489, *P* = 0.712). These data showed the negative association between serum ApoC3 levels and HBV DNA load in HBeAg (−) patients.

### Serum ALT, TG, ApoC3 and Lpa levels were correlated with HBV DNA load

We performed receiver operating characteristic (ROC) curve and chose the median value of 3.97 log HBV DNA as a cutoff point in CHB patients. In Table 2, there were no significant differences on age, BMI, AST, TBI, DBI, Chol, HDL, LDL, ApoA1, ApoB and ApoA5 between two groups (log HBV DNA < = 3.97 and >  3.97). However, the serum ALT in (log HBV DNA < = 3.97) group was significantly lower than that in (log HBV DNA >  3.97) group, and serum TG, ApoC3 and Lpa levels in (log HBV DNA < = 3.97) group were significantly higher than those in (log HBV DNA > 3.97) group. These data showed the relationship between ALT, TG, ApoC3 or Lpa and HBV DNA load.

**Table 2:** Association of Clinical and liver biochemical profiles of chronic hepatitis B with HBV DNA levels

Log HBV DNA	< = 3.97 (*n* = 248)	> 3.97 (*n* = 136)	*P*
Gender (M/F)	141/107	55/81	–
Age (years)	41.77 ± 13.68	41.27 ± 13.38	0.474
BMI (kg/m^2^)	21.98 ± 1.25	22.23 ± 1.52	0.582
ALT (U/L)	50.88 ± 2.05	66.50 ± 4.35	0.044*
AST (U/L)	44.61 ± 3.25	54.39 ± 6.10	0.079
TBI (μM)	11.52 ± 0.57	12.67 ± 0.86	0.459
DBI (μM)	5.30 ± 0.60	5.52 ± 0.64	0.801
Chol (mM)	4.44 ± 0.08	4.37 ± 0.09	0.917
TG (mM)	1.29 ± 0.06	1.09 ± 0.02	0.032*
HDL (mM)	1.29 ± 0.03	1.30 ± 0.04	0.964
LDL (mM)	2.33 ± 0.06	2.30 ± 0.08	0.975
ApoA (g/L)	1.55 ± 0.04	1.54 ± 0.05	0.826
ApoB (g/L)	0.90 ± 0.02	0.88 ± 0.03	0.794
ApoA5 (ng/mL)	100.18 ± 2.84	105.55 ± 3.69	0.607
ApoC3 (μg/mL)	53.29 ± 4.26	45.58 ± 2.64	0.045*
Lpa (mg/mL)	14.29 ± 0.75	13.19 ± 0.54	0.013*

*P < 0.05, *BMI* Body mass index

## Discussion

Chronic liver diseases can interfere with hepatic metabolism of lipids, lipoproteins or apoliproteins [16]. HBV infection is reported to be a major cause of chronic liver disease [17], which indicates the close relationship between HBV and lipid metabolism. In our study, we examined the serum levels of ALT, AST, TBI, DBI, Chol, TG, HDL-C, LDL-C, ApoA1, ApoB, ApoC3, ApoA5 and Lpa in healthy individuals and CHB patients. Biochemical test and ELISA results showed that serum ALT, AST and Chol levels were elevated, but serum TG, HDL-C, LDL-C, ApoA1, ApoB, ApoC3 and Lpa levels were decreased in CHB patients. Pearson’s correlation analysis indicated the negative association between serum ApoC3 and HBV DNA load in HBeAg (−) CHB patients. These findings provided new evidence about the effect of HBV infection on lipid metabolism and indicated the relationship between ApoC3 and HBV DNA load.

Apoliproteins exert enzyme cofactors, receptor ligands or lipid transfer carriers via binding lipids to form lipoproteins [18]. Apoliproteins family contains several sub-classes, including ApoA, ApoB, ApoC and so on. Previous study showed that plasma ApoA1, ApoB and ApoA5 levels are decreased in CHB patients [14, 19, 20]. It is indicated that HBV suppresses the synthesis and secretion of ApoA1 by inducing the hypermethylation of the ApoA1 promoter [21], inhibits the expression of ApoB via the suppression of microsomal triglyceride transfer protein [22] and blocks the levels of ApoA5 through its core gene [14]. In our study, we found that serum ApoA1, ApoB and ApoA5 levels were decreased in HBeAg (−) and HBeAg (+) CHB patients, which were in agreement with the previous research. In addition, we also found that plasma HDL-C and LDL-C levels were reduced in CHB patients. It is reported that ApoA1 is a major protein component of HDL-C [21], ApoB is the major structural protein associated with LDL-C [23], and ApoA5 is involved in elevating HDL-C [14]. Thus the decrease on HDL-C and LDL-C content might be related with the decrease on ApoA1, ApoB and ApoA5 in CHB patients. However, further experiments need to be carried out to explore the detailed mechanism.

ApoC3 is involved in regulating the plasma metabolism of triglyceride and TG-rich lipoproteins (TRL) by inhibiting the clearance of TRL particles [24]. Previous study has clearly shown that a decrease in ApoC3 is associated with a significant decrease in TG levels [25]. It is indicated that HBV down-regulated the synthesis and secretion of ApoC3 via its X gene and lower serum levels of ApoC3 and TG were observed in CHB patients [15]. In our study, we found that serum ApoC3 and TG levels in CHB patients were decreased, which is in accordance with the above results. These results provided new evidence about the physiological function of ApoC3 and confirmed its promise as a valid therapeutic target for TG-lowering. In addition, we found that serum ApoC3 level is negatively associated with the HBV DNA load in HBeAg (−) CHB patients, not in HBeAg (+) CHB patients, which suggests the function of ApoC3 is related with the status of HBeAg. HBeAg is a non-particulate version of the HBV nucleocapsid protein, which is secreted at an early stage in the HBV replication cycle [26]. It is reported that an HBeAg(−) status and intermediate HBV DNA levels are associated with severe diseases in CHB patients [27]. And the presence of steatosis is associated with various host factors and HBeAg negativity [28]. These data revealed the key role of HBeAg during HBV infection. HBeAg contributes to the establishment and persistence of chronic infection through complex inflammatory and immune response [29]. For example, HBV inhibits LPS-induced NLRP3 inflammasome activation and IL-1B production by HBeAg, not HBsAg [30]. Taken together, our study combined the lipid metabolism with immune response in CHB patients, which shed new light for HBV diagnosis and therapy. However, more experiments need to be performed to elucidate the molecular effects of HBeAg on ApoC3 expression.

## Conclusions

In summary, we mainly summarized the serum levels of Apo members (ApoA1, ApoB, ApoC3, ApoA5) in CHB patients and revealed the negative relationship between ApoC3 and HBV DNA load in HBeAg(−) CHB patients. These findings indicated the effect of HBV infection on lipid metabolism and determined ApoC3 as a serum marker to facilitate the management of CHB in clinical practice or a potential helpful agent in CHB treatment. However, there are some limitations in our study. Firstly, the number of CHB patients in the study cohort is relative insufficient. Our results need to be confirmed in a larger set of patients. Secondly, the CHB patients in our study are mainly focused on shandong peninsula. This study has certain regional specificity. Thirdly, although we revealed the negative association between ApoC3 and HBV DNA load in HBeAg (−) CHB patients, the detailed mechanism remains elusive.

## Data Availability

All data generated or analyzed during this study are included in this published article.

## Abbreviations

ALT: alanine aminotransferase
ApoA1: Apolipoprotein A1
ApoA5: Apolipoprotein A5
ApoB: Apolipoprotein B
ApoC3: Apolipoprotein C3
AST: Aspartate transaminase
BMI: body Mass index
CHB: Chronic hepatitis B
Chol: Total cholesterol
DBI: Direct bilirubin
HBV: Hepatitis B virus
HDL-C: High-density lipoprotein cholesterol
LDL-C: Low-density lipoprotein cholesterol
TBI: Total bilirubin
TG: Triglycerides
TRL: TG-rich lipoproteins
